# Supplementary material for: AffyMAPSDetector: a software tool to characterize Affymetrix GeneChip™ expression arrays with respect to SNPs
Source: BMC Bioinformatics. 2007 Jul 30;8:276. doi: 10.1186/1471-2105-8-276 (PMC1959249; doi:10.1186/1471-2105-8-276)
Supplement: Additional file 13 — AffyMAPSDetector v1 source code. AffyMAPSDetector source code. [file 1471-2105-8-276-S13.zip › AffyMAPSDetector_v1_src/doc/affychip/snpdetector/model/SnpFinder.html]

SnpFinder


|  |  |  |  |
| --- | --- | --- | --- |
| **Overview** \| **Package** \| **Class** \| **Use** \| **Tree** \| **Deprecated** \| **Index** \| **Help** | | |  |
| **PREV CLASS**   **NEXT CLASS** | **FRAMES**    **NO FRAMES**     **All Classes** |  |  |
| SUMMARY: NESTED \| FIELD \| CONSTR \| METHOD | DETAIL: FIELD \| CONSTR \| METHOD |  |  |


---


## affychip.snpdetector.model Class SnpFinder

```
java.lang.Object
  affychip.snpdetector.model.SnpFinder
```

---

public class **SnpFinder** extends java.lang.Object

Title: File Manager

Description: Given a list of SNP locations for a particular gene, this
program tell how many, if any, probes for this gene on Affy chip contain SNP
and at what location in the probe-sequence.

Copyright: Copyright (c) 2004

Company: George Mason University

**Version:**
:   1.1

**Author:**
:   Sunita Kumari

---

**Constructor Summary**

`SnpFinder()`


|  |  |
| --- | --- |
| **Method Summary** | |
| `static void` | `main(java.lang.String[] args)` |
| `static Probe` | `parseProbe(java.lang.String line)`             probe:HG-U95Av2:1000\_at:399:559; Interrogation\_Position=1367; Antisense;TCTCCTTTGCTGAGGCCTCCAGCTT |

**Methods inherited from class java.lang.Object**

`clone, equals, finalize, getClass, hashCode, notify, notifyAll, toString, wait, wait, wait`

**Constructor Detail**

### SnpFinder

```
public SnpFinder()
```


**Method Detail**

### parseProbe

```
public static Probe parseProbe(java.lang.String line)
```

:   probe:HG-U95Av2:1000\_at:399:559; Interrogation\_Position=1367; Antisense;TCTCCTTTGCTGAGGCCTCCAGCTT

    :   **Parameters:**: `line` - **Returns:**

---


### main

```
public static void main(java.lang.String[] args)
```


---


|  |  |  |  |
| --- | --- | --- | --- |
| **Overview** \| **Package** \| **Class** \| **Use** \| **Tree** \| **Deprecated** \| **Index** \| **Help** | | |  |
| **PREV CLASS**   **NEXT CLASS** | **FRAMES**    **NO FRAMES**     **All Classes** |  |  |
| SUMMARY: NESTED \| FIELD \| CONSTR \| METHOD | DETAIL: FIELD \| CONSTR \| METHOD |  |  |


---
